# Uncovering the potential of evaluative conditioning in shaping attitudes toward sustainable product packaging

**DOI:** 10.3389/fpsyg.2024.1284422

**Published:** 2024-03-14

**Authors:** Nikki Leeuwis, Tom van Bommel, Manos Tsakiris, Maryam Alimardani

**Affiliations:** ^1^Department of Cognitive Science and Artificial Intelligence, Tilburg School of Humanities and Digital Sciences, Tilburg University, Tilburg, Netherlands; ^2^Unravel Research, Utrecht, Netherlands; ^3^Department of Psychology, Royal Holloway, University of London, Egham, United Kingdom; ^4^Centre for the Politics of Feelings, School of Advanced Study, University of London, London, United Kingdom

**Keywords:** evaluative conditioning, affective images, sustainability, attitude change, implicit associations

## Abstract

**Introduction:**

The necessity to promote pro-environmental behavior change in individuals and society is increasingly evident. This study aimed to investigate the effect of evaluative conditioning on consumers’ perception of product packaging.

**Methods:**

We first produced two stimulus sets: one including images of supermarket products with different packaging and the other containing affective images of healthy nature (positive) and climate change impact (negative). These images were then paired in an evaluative conditioning experiment where respondents were informed about the impact of product packaging.

**Results:**

We found an effect of conditioning depending on the initial sustainability perception that participants had toward product packaging. Pairing products for which participants were uncertain about their sustainability with negative or positive affective images had a significant effect on the sustainable associations of the consumers in a negative or positive direction, respectively. However, the impact of conditioning on products that clearly had (un)sustainable packaging was not that strong.

**Discussion:**

These results provide new tools and evidence to further investigate the power of evaluative conditioning in pro-environmental attitude and behavior change.

## 1 Introduction

The behavior of people on a daily basis has an effect on their own health and well-being, but also on the health and well-being of other individuals, groups, and on society at large, which thereby causes and alleviates social problems such as climate change (Fishbein and Ajzen, 2010). The effects of human behavior on the environment have been unequivocally established by the Intergovernmental Panel on Climate Change; humans have warmed the atmosphere, oceans and land. Widespread and rapid changes in the atmosphere, ocean cryosphere, and biosphere have occurred (SPM, p. 5). Amongst other factors, pollution from fossil-based plastic waste and other waste related to packaging have a devastating effect on the quality of air, soil, and water, which accelerates climate change (Borrelle et al., 2020; Boz et al., 2020; Phelan et al., 2022).

Although the majority of people say they prefer sustainable and waste-free products (Rokka and Uusitalo, 2008), they do not always purchase them (Jerzyk, 2016) and thus excessive consumption patterns still exist (Stolz et al., 2013). This discrepancy between what people say and what they do is labeled as the attitude-behavior gap (Kennedy et al., 2009). This problem is often encountered when investigating ethical, social, or responsible consumer behavior. Even for people who intend to act sustainably, survey studies showed that they could not correctly describe an ecological-friendly packaging or did not have a clear idea of what it looked like (Lindh et al., 2016a,b).

Recently, Leeuwis et al. (2022b) proposed a framework for the design of behavior change interventions that could promote pro-environmental attitude and behavior among consumers. In their review, they pointed out that behavior change interventions could possibly rely on visualizations of climate change impact, since these have been shown to induce emotional response (O'Neill and Nicholson-Cole, 2009; Lehman et al., 2019; Dal Fabbro et al., 2021) and engagement (O’Neill, 2020) in viewers. Using climate change visualization, interventions can be designed to draw attention to sustainable products (Van der Laan et al., 2017; Ischen et al., 2022) or condition consumers toward more positive emotions and reward associations in response to green products (Leeuwis et al., 2022b). This method, which is known as evaluative conditioning (also affective or emotional conditioning), has been identified as a potential method that could change consumers’ behavior toward more sustainable purchase decisions and therefore bridge their attitude-behavior gap.

Evaluative Conditioning (EC) is a form of associative learning that can be used for changing preferences by creating a relation between actions and emotional responses (Eder et al., 2019; De Houwer and Hughes, 2020). EC has been successfully implemented in several studies for behavior change in the health domain, although the results on the lasting effects of the intervention have not been fully conclusive (Houben et al., 2010; Hollands et al., 2011; Hollands and Marteau, 2016; Papies, 2017; Moran et al., 2023). Successful examples include pairing healthy food with images carrying positive affect (Halbeisen and Walther, 2021) or unhealthy food with aversive images (Hollands et al., 2011), after which the preferences for products changed and participants were more likely to pick a piece of fruit instead of the snack they would have chosen before the conditioning. EC has also been used to promote pro-vaccination attitudes: aversive cues (e.g., images showing sickness or death) in ads promoting flu vaccine products could enhance attitudes towards a co-occurring vaccine brand, but only when people were under a low attentional load (Fan et al., 2021).

Especially following the latter example, ethical concerns regarding EC are raised whether the presentation of affective imagery impairs the autonomy with which individuals make their choices (Biegler and Vargas, 2016). EC is very common in the real world where commercial products are paired with affective images to improve brand attitude, which follows from studies on EC with consumer products (Pleyers et al., 2007; Sweldens et al., 2010). Usually, EC in advertising is aimed at promoting brand attitudes, however, companies might be tempted to use it as a technique for greenwashing; an activity in which a product is advertised to have more sustainability qualities than it actually does (Walker and Wan, 2012). This activity of greenwashing calls for a deeper understanding of the conditions that cause EC effects to take hold, both to promote sustainable products and intervene against unsubstantiated claims in favor of non-sustainable alternatives (Fernandes et al., 2020). In the line of conditioning for ethical decisions, EC has been applied to combat positive attitudes towards alcohol consumption: after the intervention, participants showed more negative attitudes toward beer, experienced less craving, and consumed less both in the lab during the taste test and outside the lab during the week following the session (Houben et al., 2010).

In the context of environmental research, studies have shown that conditioning could be an interesting intervention to explore. EC using images of cheerful animals motivated participants to perform more pro-environmental efforts, although only in half of the studies (Lange and Dewitte, 2023). Moreover, images of nature could motivate pro-environmental behavior (Yu et al., 2023) and might inform the consumer about the sustainability of products in the supermarket. For example, Meijers et al. (2021) presented images or texts concerning natural scenes when people grabbed a product in a virtual reality (VR) supermarket. This affected their attitudes as well as self-reported buying behavior toward more pro-environmental choices up to 2 weeks after the intervention. To date, there have been very few investigations into the relationship of consumers with sustainable packaging and the effect of pro-environmental interventions. This is mainly due to the lack of established datasets of images, both for the stimuli that the behavior change intervention acts on (i.e., the products) and the affective images that would be shown during the intervention (i.e., climate change visualization).

**Figure 1:**
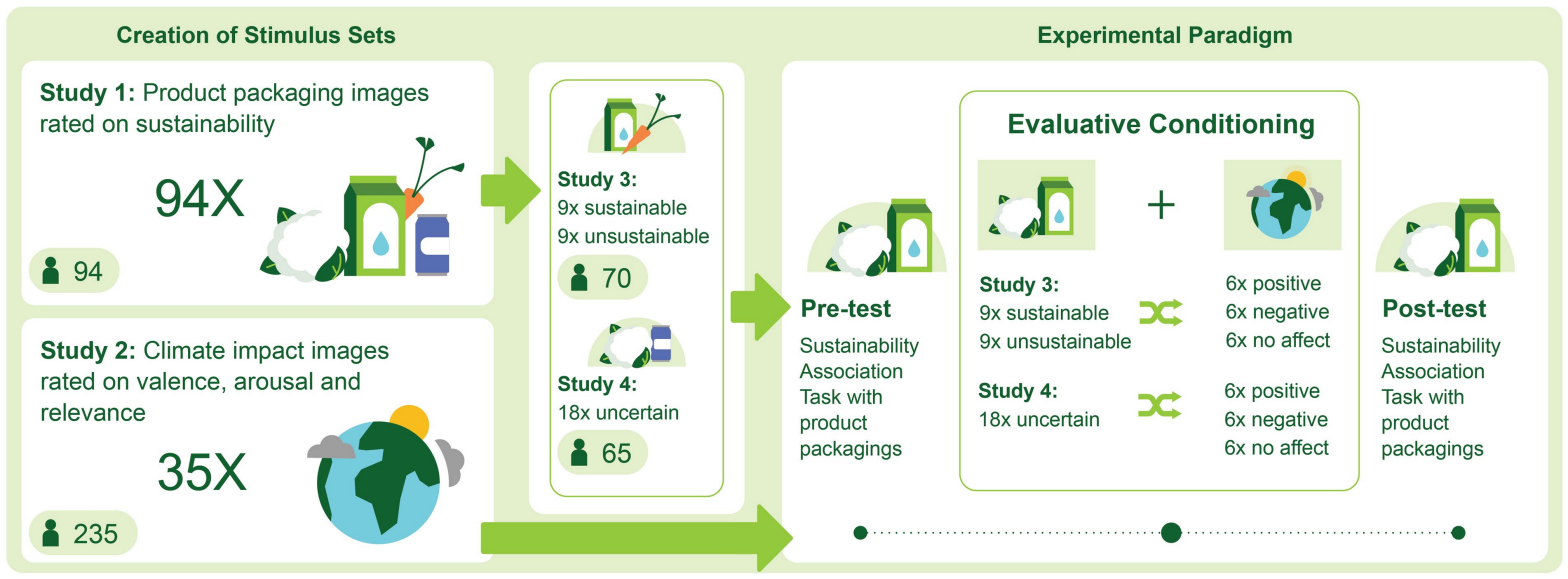
An overview of the studies presented in the manuscript. Two stimulus sets were created in Study 1 and 2, which were applied in an evaluative conditioning paradigm in Study 3 and 4. Study 1 collected sustainability ratings of supermarket product images in terms of packaging, and Study 2 collected valence ratings of nature and climate change-related images. Consequently, the nature image pairs that were most strongly divided in valence were coupled to the product packaging images as evaluative conditioning. In Study 3, product images with the highest and lowest ratings of packaging sustainability were paired with either positive, neutral, or negatively-valenced climate images. In Study 4, the same climate impact images were applied in the conditioning paradigm but this time they were paired with product images whose packaging was rated in the middle of the sustainability spectrum, i.e., participants were uncertain about their sustainability.

We here aimed to investigate the effect of evaluative conditioning on pro-environmental attitude in two steps. First, we collected and validated two stimulus sets including (1) images of supermarket products that were rated based on the sustainability of their packaging, and (2) images of nature and climate change impact that were rated based on their relevance to climate change, as well as the arousal and valence they evoked in the participants. These two image datasets together with their ratings are shared in the Supplementary material with the aim to promote collaboration and future research within this domain. Secondly, we used the collected stimulus sets to investigate the effect of evaluative conditioning on consumers’ perception of product packaging. Therefore, the work presented in this paper is divided in two parts, each consisting of two studies; In Study 1 and 2, the creation of the two stimulus sets (supermarket products and climate-related images) and the surveys pertaining to their validation are reported. In the subsequent Study 3 and 4, we present the outcome of evaluative conditioning attempts using these stimulus sets. In Study 3, we used the climate-related affective images from Study 2 to condition participants toward product packaging that, based on the ratings of Study 1, was rated on the extremes of sustainability scale (i.e., clearly sustainable or unsustainable). In Study 4, we repeated this conditioning for product packaging that was rated in the middle of the scale (i.e., their sustainability was ambiguous to the participants). An overview of the studies is presented in Figure 1.

## 2 Creation of stimulus sets

The creation of validated stimulus sets for a conditioning paradigm was conducted in two studies. In the first study, images of products in a supermarket were collected and participants rated their sustainability on a 7-point Likert scale. In the second study, images of nature and climate change were rated on a 9-point Likert scale of relevance, arousal, and valence. Their methods and results will be discussed separately.

### 2.1 Study 1: Product images

#### 2.1.1 Methods

##### 2.1.1.1 Stimuli

Images were selected based on their appearance on a grocery retailer website^1^ and websites with open creative licenses such as Pexels.com. They were selected based on their packaging; they either contained no packaging, re-usable/recyclable packaging, or packaging that claimed to be better for the climate or packaging in excessive plastic. The images from the websites were modulated such that their background was white and the logos on all packages were made unrecognizable. In total, 94 images were included in the test, which can be found in the Supplementary material A.

##### 2.1.1.2 Participants

A total of 94 participants (21 Male, 73 Female; *M_age_* = 21.16, *SD_age_* = 2.92) were recruited using the university subject pool. They received course credit in return for their participation in the experiment. The study was approved by the Research Ethics Committee of Tilburg School of Humanities and Digital Sciences (TSHD_RP123a). Prior to the experiment, participants read an information letter and signed an informed consent form. Of these respondents, 26 were not responsible for the grocery shopping themselves (37.7% of the females, 40% of the males). The number of respondents was determined following the pilot study (*n* = 68) of Koenig-Lewis et al. (2022), where products were rated on a Likert scale to determine their perceived healthiness, and then oversampled.

##### 2.1.1.3 Questionnaires

Three questionnaires were collected prior to the task; demographics, New Environmental Paradigm, and Health Consciousness. The demographical questions assessed the gender and age of the participants as well as their responsibility for grocery shopping at least for the majority of their meals. The New Environmental Paradigm (NEP; Dunlap et al., 2000) is a 15-item questionnaire that assesses environmental beliefs and is answered on a 5-point Likert scale. The odd questions of the NEP are worded in line with pro-ecological view and hence are reversed when calculating the average score for each participant. Health Consciousness (HC) was measured by 4 items on a 7-point Likert scale following Mai and Hoffmann (2015). Health is considered important for grocery purchase decisions (Koenig-Lewis et al., 2022).

##### 2.1.1.4 Procedure

**Figure 2:**
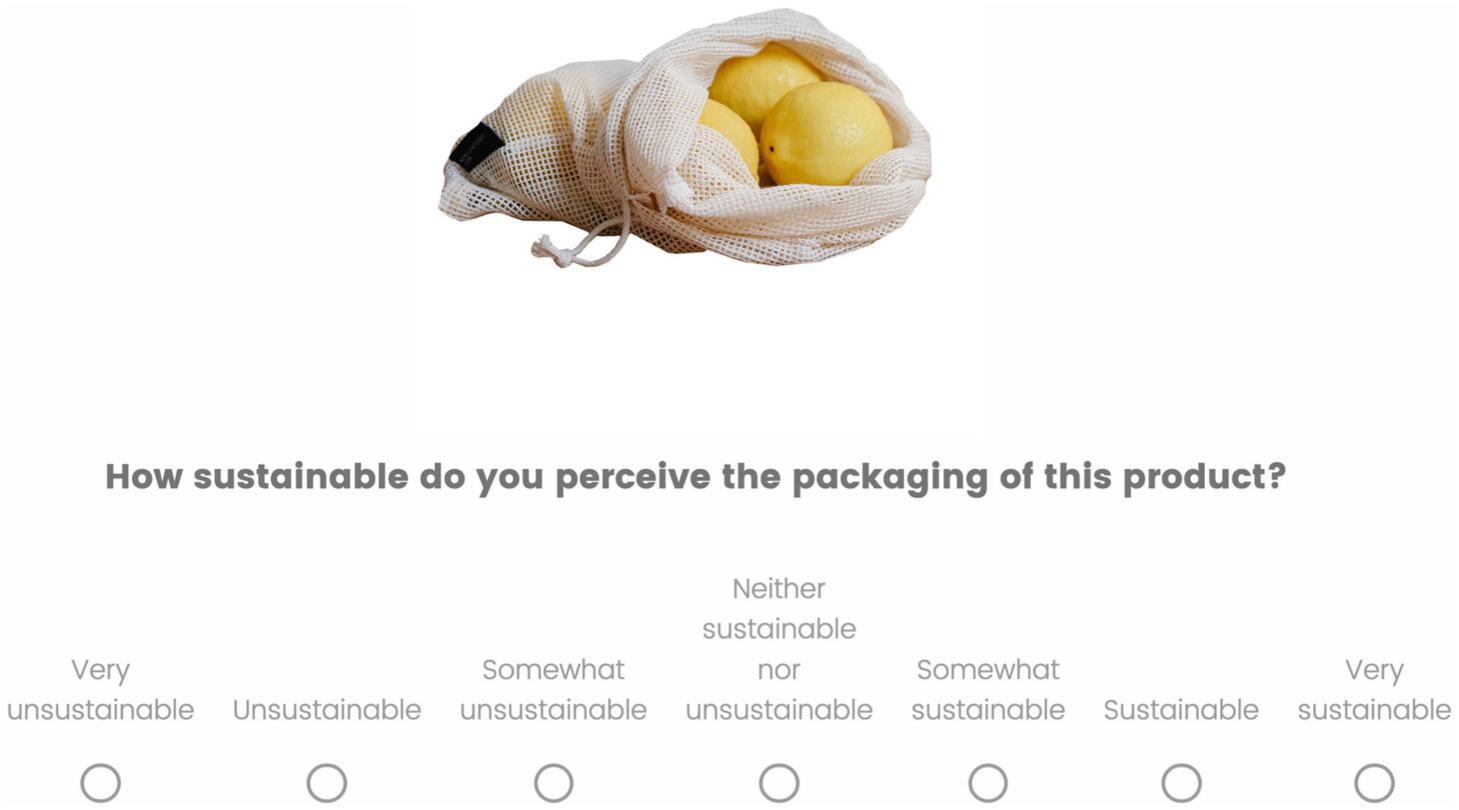
The rating task where participants rated the sustainability of the packaging of the product on a 7-point Likert scale. The direction of the scale was randomized between participants, such that for half of them “Very sustainable” was presented on the right end of the scale, and for the other half of the sample “Very sustainable” was on the left end of the scale. Moreover, within participants the same image was rated twice: once they were asked about the sustainability (as in the example here) and once about the unsustainability of the product packaging. Image reproduced from Pexels.

The survey was administered using Qualtrics. Participants read the information letter and were only able to continue when they provided informed consent. First, participants answered demographical questions and the NEP and HC questions. After that, they were introduced to the task. The task was to judge the product images on (un)sustainability on a 7-point Likert scale (Figure 2). Sustainability in this case was defined: “in the sense of packages owning attributes aiming at reducing the product’s environmental footprint. Think of the materials of the package, recyclability and ecological footprint.” The questions were repeated twice for each product; participants rated each product on both ‘sustainability’ and ‘unsustainability’ terms. Moreover, the scales were randomized between subjects: half of participants rated on a scale where *Very sustainable* was presented on the left and *Very unsustainable* on the right, whereas for the other half it was the other way around. These design choices were implemented to overcome positivity bias and any biases from left/right associations (Weijters et al., 2013). Participants rated 94 products in random order in two blocks (a total of 188 trials). Before the question changed to (un)sustainable, participants had a break. When they finished both blocks, participants were debriefed.

#### 2.1.2 Results

The product images and their mean ratings can be found in Supplementary material A.

The average NEP score was 3.60 (*SD* = 0.46) with Cronbach’s alpha of 0.76 on this 15-item questionnaire. The average Health Consciousness was 5.46 (*SD* = 0.92), and Cronbach’s alpha was 0.77 on the 4 items.

The average product packaging rating was 4.06 (*SD* = 2.17). There was no correlation between people with a higher environmental belief and their perception of product packaging sustainability (Spearman *rho* = −0.13, *p* = 0.21). Gender had no impact on product sustainability ratings [Wilcox *W*(16.48) = 753, *p* = 0.91].

There was no effect of left/right associations [*W*(47.25) = 4,169, *p* = 0.51], i.e., the direction of response options in the Likert scale (where unsustainable was presented either on the left or on the right) did not impact the ratings. Moreover, the difference between the (un)sustainable questions was not significant [*W*(47.25) = 4415.5, *p* = 0.996]: the answers on the questions “How sustainable do you perceive the packaging of this product” were not significantly higher than the question “How unsustainable do you perceive the packaging of this product.”

**Figure 3:**
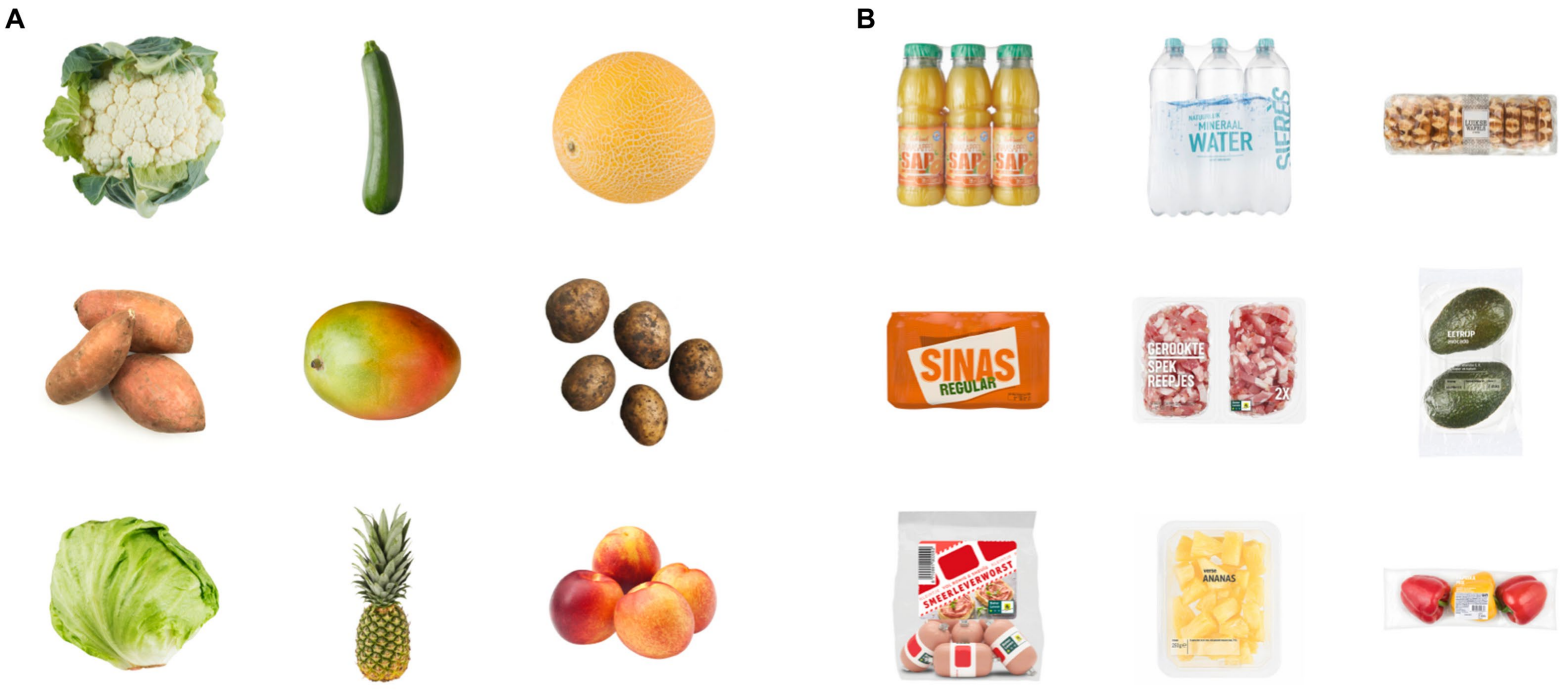
Product images that were rated on the extremes of the sustainability scale. **(A)** Product images that were rated the highest on sustainability did not include any packaging, while **(B)** products images of which the packages were rated most unsustainable used extra plastic. These 18 images were implemented in Study 3 as extreme (un)sustainable packaging images. Images reproduced with permission of Albert Heijn. Images of potatoes in 3 **(A)** reproduced from Pexels.

Products with the highest sustainability ratings did not include any packaging, while the products with the lowest sustainability ratings were packed in more plastic than what is strictly essential for containing, transporting and preserving the product (Figure 3).

#### 2.1.3 Discussion

In this study, the perception of packaging of products in the supermarket was investigated. This revealed that products without any packaging were perceived most sustainable while products packed in plastic were perceived the least sustainable. These images and their ratings were aimed to create a stimulus database for future researchers. For example, future research could use these stimuli as a baseline for interventions targeting consumers to consider waste-free product packaging more.

The images in this stimulus set were not explicitly matched in terms of product type or visual appearance. Since consumer behavior is performed on a daily basis and under high impact of external factors such as packaging visuals, texts and sizes (Orth and Malkewitz, 2008; García-Madariaga et al., 2019), it was important to incorporate as much ecological validity in the stimulus set as possible. In order to provide images that match reality as close as possible, we only removed the branding but kept packaging shapes, colors and product orientations intact. Although there were some identical products with different packaging in the initial dataset (such as cauliflower with and without packaging), these did not come forward as the strongest (un)sustainable products (which are shown in Figure 3). This led to the top and bottom products not being visually similar but instead providing the most extreme (un)sustainability contrast between packaging according to the sample.

### 2.2 Study 2: Affective images of nature

#### 2.2.1 Methods

##### 2.2.1.1 Stimuli

Nature images promoting either positive or negative valence were gathered from three sources: (1) an openly available database of affective climate change images by Lehman et al. (2019) where images were rated on relevance, arousal and valence on a 9-point Likert scale, (2) an openly available database of positive nature images by Dal Fabbro et al. (2021) that were rated on arousal and valence on a 1–9 Likert scale containing emoticons, and (3) images that were obtained from websites with creative licenses such as Pexels.com and Unsplash.com. Search terms such as ‘waste’ and ‘pollution’ were used to search for negative images. When a suitable image was found, a positive search term for that image was also used. For instance, if the selected negative image showed a polluted ocean, we would search for ‘ocean’ and ‘water’ to find its positive counterparts in the same context.

For images to be considered, we adhered to the following guidelines: the images should not contain text, people and graphs, signs, or otherwise designed stimuli. Following these inclusion criteria, we selected images from Lehman et al. (2019) with a valence rating below 4 as negative images and those with a valence rating above 6 as positive images. Similarly, from images of Dal Fabbro et al. (2021), we considered positive images with a valence above 7.4. For each context shown in these images, the image with the highest valence score was selected, but only if there was a negative contextual counterpart (i.e., the beach was selected when an image of a beach with waste was included).

From this initial selection of nature images, pairs of positive and negative images were further defined for the validation survey based on their source database ratings or our own interpretation when no rating was available. It was important to have contextually matched positive and negative images as the planned intervention in Study 3 and 4 conditioned the products in both directions. There was a clear representation of waste-related images in these defined pairs as they show the most direct effect of plastic packaging on the environment. For a few pairs, there were multiple positive images from the database that were fit for pairing; these were all included in the validation survey so that participants could choose the image to which they had the strongest positive reaction.

Moreover, 10 neutrally-valenced images (the 10 images that scored on the middle of the Likert scale for valence) from the database of Lehman et al. (2019) were included as distractors to ensure that participants’ answers were not biased toward the extremes. In total, 35 images were included for validation (11 negative, 10 neutral and 14 positive images). The images included can be found in Supplementary material B.

##### 2.2.1.2 Participants

In total, 235 respondents (129 Male, 104 Female, 2 Non-binary, *M_age_* = 53.9, *SD_age_* = 20.0) were recruited using a panel agency. They received a small monetary reward in return for their participation in the experiment. The study was approved by the Research Ethics Committee of Tilburg School of Humanities and Digital Sciences (TSHD_RP123a). Prior to the experiment, respondents read an information letter and signed an informed consent form. The number of respondents was determined following Lehman et al. (2019), who conducted their study on 67 respondents, and Dal Fabbro et al. (2021) where the number of ratings per image was between 36 and 108. We decided to take the high aim of 108 ratings per image, which was consequently oversampled to an average of 130 ratings per image.

##### 2.2.1.3 Questionnaires

**Figure 4:**
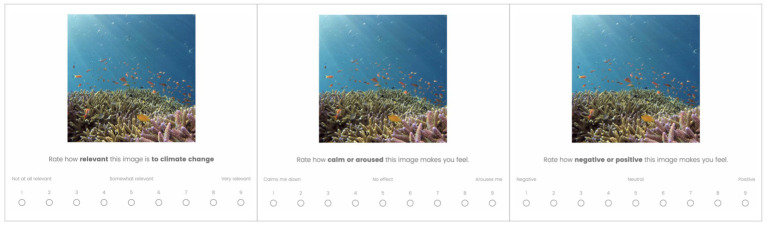
The rating task where subjects rated the relevance, arousal and valence they felt evoked by the image on a 9-point Likert scale. Images reproduced from Unsplash.

As in Study 1, demographics and the New Environmental Paradigm (NEP) were assessed. Images were rated on a 9-point Likert scale regarding the relevance, arousal and valence it evoked with the participant (Figure 4). The relevance of the image to climate change asked how strongly the picture was related to global warming, concerning both positive and negative relevance (e.g., if a positive scenery is very relevant to climate change, participants were instructed to rate a 9/9). Arousal was defined as how calm or aroused participants felt when watching the image. Arousal in this case referred to the strength of the participants’ gut reaction to the image and served as a measure of stimulation or frustration elicited by the image. The valence was measured as the negative or positive emotion participants felt when watching the image. They were asked whether the image made them happy or sad/angry.

**Figure 5:**
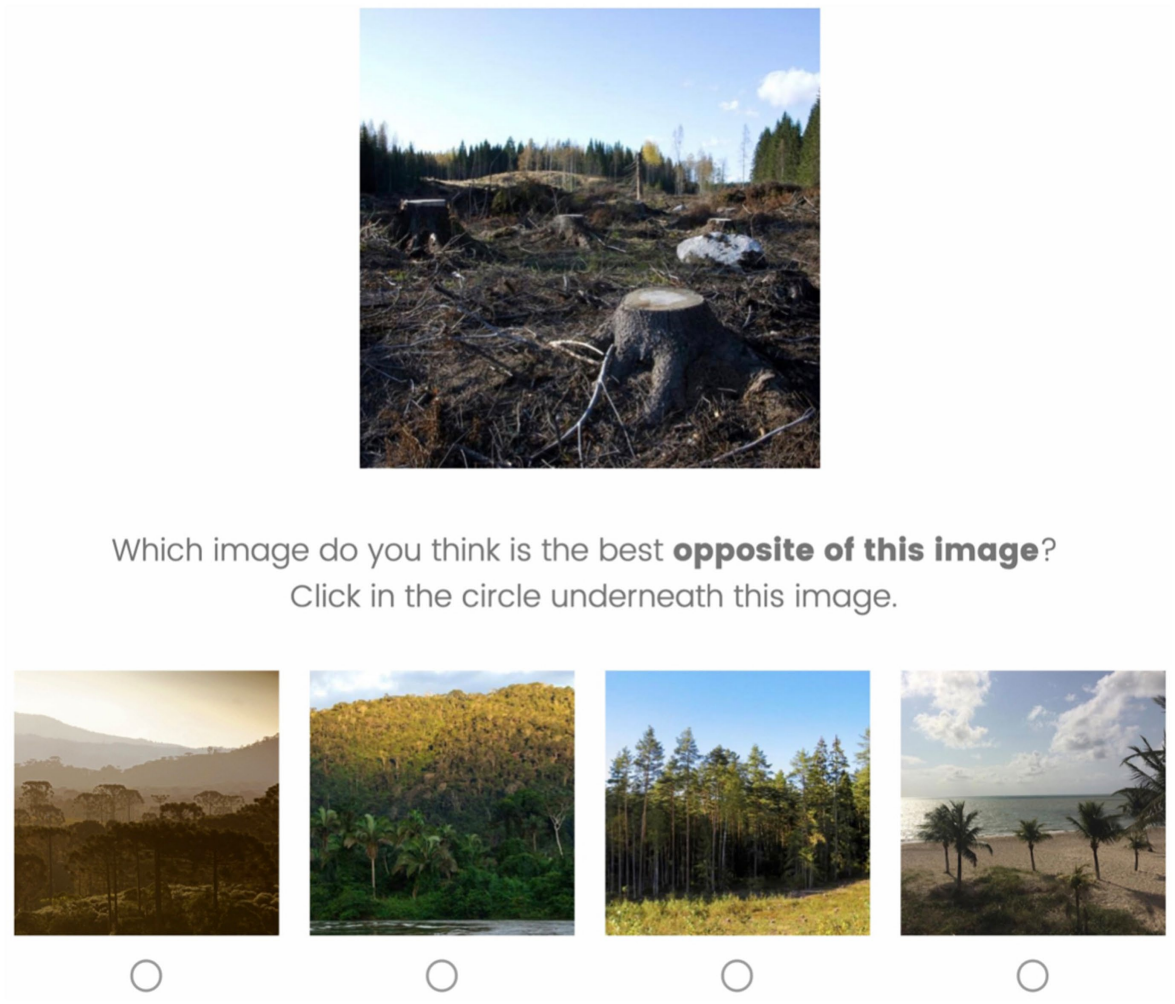
The pairing task where participants chose the image they thought was the best positive counterpart of the negative image shown. Image (top) reproduced under the terms of CC-BY 4.0 from Lehman et al. (2019). Images (bottom) from left to right reproduced with permission from e-NatPOEM (Dal Fabbro et al. 2021) image 099, image 064, Unsplash and e-NatPOEM (Dal Fabbro et al. 2021) image 393.

Lastly, participants performed a pairing task where a negative image was presented together with four neutral or positive images of which the participant had to choose one that in their view was the best positive counterpart of the negative image in terms of visual and conceptual objects (Figure 5). This pairing task served to identify the most appropriate matches in participants’ opinions as for some negative stimuli, multiple positive counterparts were hypothesized by the researchers in the selection phase.

##### 2.2.1.4 Procedure

The survey was administered using Qualtrics. Participants read the information letter and were only able to continue when they agreed to the informed consents. First, participants performed the rating task where they evaluated a random subset of images (20 out of 35, in random order) on their valence, arousal and relevance to climate change on a 9-point Likert scale. Afterwards, participants performed a pairing task where negative images were presented together with four neutral or positive images that visually or contextually resembled them. Participants were instructed to choose one of the four images that they considered the most appropriate opposite of the shown negative image. To minimize fatigue and loss of attention, each participant rated a random subset of pairs (6 out of 11 pairs that were included in the study). Lastly, they answered demographical questions and the NEP questions. After that participants were debriefed.

#### 2.2.2 Results

The selected climate-related images and their mean ratings can be found in Supplementary material B.

On average, images were rated by 130.56 participants (*SD* = 2.74, *min* = 125, *max* = 136). On average, the images were rated 5.90 (*SD* = 0.94) on relevance to climate change, 5.55 (*SD* = 0.96) on arousal and 4.93 (*SD* = 1.41) on valence.

The neutral images were rated slightly above the scale mean of five points: 5.35 (*SD* = 0.47) on valence [*t*(9) = 2.35, *p* = 0.04], although not significant on relevance [*M* = 5.13, *SD* = 0.70, *t*(9) = 0.60, *p* = 0.56], and arousal [*M* = 5.18, *SD* = 0.34, *t*(9) = 1.62, *p* = 0.14]. Negative images (valence *M* = 3.04, *SD* = 0.38) were significantly more arousing (*M* = 6.63, *SD* = 4.86) than positive images (valence *M* = 6.02, *SD* = 0.71, arousal *M* = 4.86, *SD* = 0.34) (*t*(20.55) = 11.74, *p* < 0.001, *CI* = [1.46, 2.09], *d* = 4.37). Moreover, negative images were perceived as more relevant to climate change (*M* = 6.83, *SD* = 0.47) than positive images (*M* = 5.31, *SD* = 0.63) (*W* = 293, *p* < 0.001, *CI* = [1.10, 1.97], *d* = 0.752).

The average NEP in the sample was 3.68 (*SD* = 0.54), Cronbach’s alpha for this 15-item scale was 0.82. NEP was negatively correlated with average valence ratings per participant (*M* = 4.93, *SD* = 0.96) (Spearman *rho* = −0.332, *p* < 0.001, *CI* = [−0.44, −0.21]), indicating that participants with higher environmental belief on average rated all images lower on valence. A positive correlation was observed between NEP and the average relevance rating per participant (*M* = 5.90, *SD* = 1.47) (Spearman *rho* = 0.162, *p* = 0.013, *CI* = [0.04, 0.28]), indicating that participants with higher environmental belief also rated the images to be more relevant to climate change. For arousal, no such an effect was observed.

**Figure 6:**
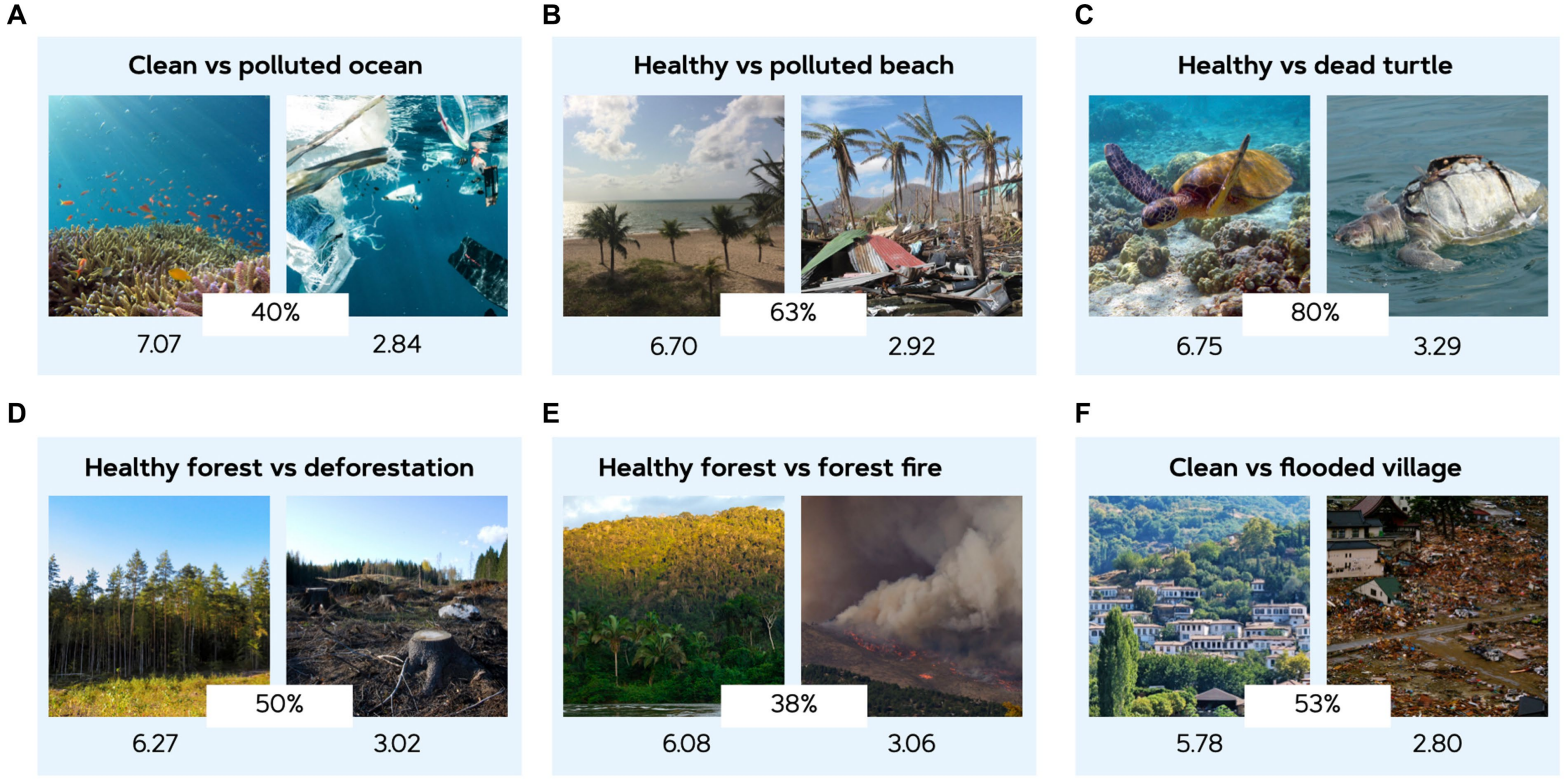
Pairs of affective (positive and negative) images that were selected in Study 2 based on their conceptual match as perceived by participants and the valence difference between them. These pairs are used in the following studies for evaluative conditioning. The mean valence score is provided below each image (on a 9-point Likert scale), with the most negative image being rated 2.80 (where 1 was the minimum value to be selected) and the most positive image being rated 7.07 (where 9 was the maximum value that could be selected). These image pairs served as conditioning stimuli in the consequent Study 3 and Study 4. **(A)** Images reproduced from Unsplash (1,2). **(B)** Images reproduced with permission from e-NatPOEM (Dal Fabbro et al. 2021) image 393 and under the terms of CC-BY 4.0 from Lehman et al. (2019). **(C)** Images reproduced under the terms of CC-BY 4.0 from Lehman et al. (2019). **(D)** Images reproduced from Unsplash and under the terms of CC-BY 4.0 from Lehman et al. (2019). **(D)** Images reproduced with permission from e-NatPOEM (Dal Fabbro et al. 2021) image 064 and from Unsplash. **(E)** Images reproduced under the terms of CC-BY 4.0 from Lehman et al. (2019).

For all images, the highest match perceived by the participants on average was 50.05% (*SD* = 13.10%), where the best match was between the positive and negative image of the turtle (79.84%) (Figure 6) and the worst match between hills of garbage and a healthy forested hill (35.93%), but this mainly had to do with the number of similar options available.

Consequently, from all climate-related images, six pairs (positive vs. negative) were selected for evaluative conditioning to be used in Study 3 and 4. These six pairs of images can be seen in Figure 6. The values under images present their valence, and the values in between images indicate the match rate of the pair and their difference in arousal. The selection was based on participants’ pairing and how large the difference in valence ratings were. The average difference in valence evoked by this selection of pairs was 3.45 (*SD* = 0.48), where the average valence rating for positive images was 6.44 (*SD* = 0.48) and for negative images was 2.99 (*SD* = 0.18). The arousal (*M* = 4.73, *SD* = 0.27) and relevance (*M* = 5.50, *SD* = 0.57) ratings for positive images were lower than the arousal (*M* = 6.87, *SD* = 0.10) and relevance (*M* = 6.98, *SD* = 0.11) ratings for the negative images. On average, the match between the selected pairs as perceived by the participants was 53.95% (*SD* = 15.54%), meaning that almost half of the participants selected the positive image in these pairs as being the best counterpart to the negative image presented (out of four choices).

#### 2.2.3 Discussion

This study aimed to validate the climate-related images that were presented online and classify them into pairs of positive and negative affect. Most pairs were matched by participants as we expected, there were only two images that were matched by the participants differently: the healthy forest (Figure 6E, left image) that we picked as possible match for the deforestation (Figure 6D, right image) served better in combination with the forest fire (Figure 6E, right image). Moreover, one of the neutral images (Figure 6F, left image) was perceived as the best positive counterpart for the image of the flooded village (Figure 6F, right image). The valence of this image was also perceived slightly above average and therefore created sufficient difference between positive and negative valence rating for this image pair.

Results were comparable with Lehman et al. (2019); they showed NEP scores indicating environmental attitude were correlated to the perceived relevance of the image to climate change, which was also observed in our sample. Moreover, negative images in the study of Lehman et al. (2019) were perceived more arousing in general, which is consistent with our findings.

The climate-related affective images collected and validated in this study can help future studies in in investigating environmental psychology and behavior. For example, researchers can use these images to evaluate how emotions and attitudes towards climate change can impact pro-environmental behavior. In conditioning paradigms, such a database could help improve interventions targeting the emotional component of pro-environmental behavior. Additionally, the images could be combined with text for example to examine responses to climate change information in the media.

## 3 Experimental validation

With the stimulus sets defined in Study 1 and 2, we pursued to examine the effect of evaluative conditioning as an intervention for pro-environmental attitude change. We did this in two steps; the first experiment (Study 3) aimed at exploring the impact of conditioning on products at the extremes of the sustainability scale, meaning that participants clearly perceived their packaging qualities to be either sustainable or unsustainable. The second experiment (Study 4) aimed at exploring the impact of conditioning on product images that received a rating around the midpoint of the sustainability scale, which meant there was no consensus about how (un)sustainable the packaging was.

The methods of both studies were identical, only the product images were different, but they were paired with the same climate impact images. The data collection for the two studies was conducted in two phases in order to reduce the number of trials and the amount of time required from the participants. In this chapter, we first report the similarities and differences in methods of Study 3 and 4, and then present their results together in the Results section to enable comparison.

### 3.1 Methods

We first describe the general methods for evaluative conditioning (sampling, procedure, questionnaires, tests, and analysis), then present the stimuli in each study and finally in the last subsection we report the analysis of both studies.

#### 3.1.1 Participants

Before the experiment, a power analysis was conducted using G*Power (Faul et al., 2007) to determine the number of required participants in the study. The power was set at 0.95, the alpha level at 0.05, and the effect size of the primary outcome at *d* = 0.50, which followed a review on evaluative conditioning (Hofmann et al., 2010) and was used by Hollands et al. (2011). With the main analysis using a three-group ANOVA test, the required sample size would be 54 participants. In both studies, this number was oversampled (70 participants in Study 3 and 65 participants in Study 4) to ensure that after data rejection (see section Analysis of SAT) the sample size would still be sufficient. Participants were recruited from the university sample pool. They were all university students and received course credit in return for their participation in the experiment. The study was approved by the Research Ethics Committee of Tilburg School of Humanities and Digital Sciences (TSHD_RP123a). Prior to the experiment, participants read the information letter and signed the informed consent form.

##### 3.1.1.1 Study 3

For Study 3, a total of 70 respondents (17 Male, 51 Female, 2 Non-binary; *M_age_* = 20.74, *SD_age_* = 3.44) were recruited to participate in the online experiment. After preprocessing the data (see section Analysis of SAT for the exclusion criteria), 4 respondents were removed, leaving 66 participants in the analysis (16 Male, 49 Female, 1 Non-binary; *M_age_* = 20.58, *SD_age_* = 3.10). This sample size provided 80% power to detect an effect size of *r* = 0.350 or greater in a paired *t* test with a 5% false-positive rate.

##### 3.1.1.2 Study 4

In Study 4, 65 respondents (25 Male, 40 Female; *M_age_* = 20.23, *SD_age_* = 2.65) were recruited to participate. After preprocessing the data (see section Analysis of SAT for the exclusion criteria), 4 respondents were removed, leaving 61 participants in the analysis (22 Male, 39 Female; *M_age_* = 20.26, *SD_age_* = 2.71). This sample size provided 80% power to detect an effect size of *r* = 0.365 or greater in a paired *t* test with a 5% false-positive rate.

#### 3.1.2 Procedure

**Figure 7:**
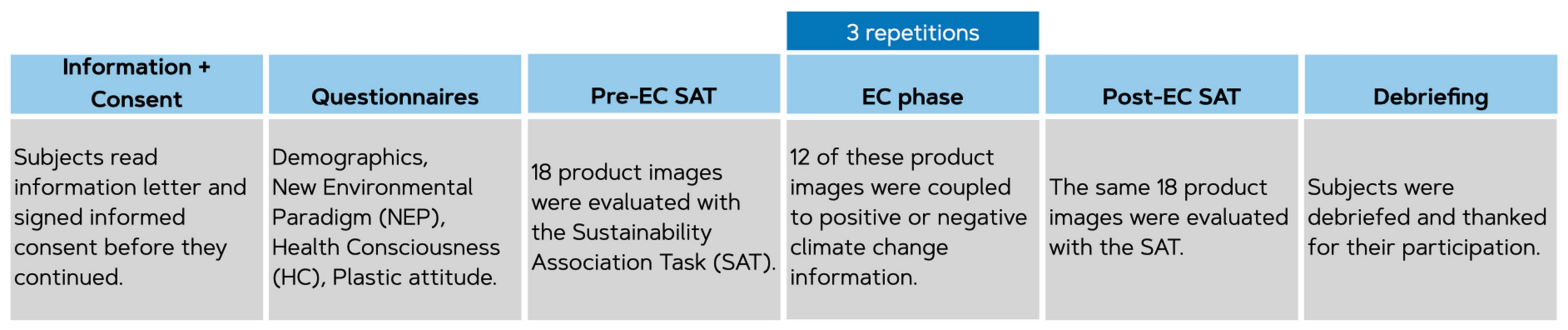
Overview of the procedure in Study 3 and 4.

The experiments were administered online using Qualtrics and could only be taken on desktop. An overview of the procedure is visualized in Figure 7. Participants read the information letter and were only able to continue when they signed the informed consent. First, demographics, New Environmental Paradigm (NEP; Dunlap et al., 2000), Health Consciousness (HC: Mai and Hoffmann, 2015; Koenig-Lewis et al., 2022) and attitude towards plastic packaging were assessed. After that, participants conducted the Sustainability Association Task (SAT) on 18 product images. This was followed by Evaluative Conditioning (EC) phase where participants received affective information about the products and their packaging via climate-related images that were highly positive or negative on valence ratings. Positive information was provided for six products and negative information for six other product images (note that six products were not included in the conditioning phase at all in order to serve as control items). Each product image was shown for 1 s, followed by the climate image for 1 s. Then a fixation cross was shown with an inter-trial interval that varied between 800 and 1,200 ms before the next combination was shown. All combinations were shown three times (in three blocks) to ensure associative learning. In between every block, a grey circle was shown where the participant could choose to take a break and continue when they felt ready to do so. Once all three blocks were completed, the SAT was once again conducted to evaluate attitude change. Afterwards, participants were debriefed that the presented image combinations were not real but rather served as experimental manipulation. Finally, participants were thanked for their participation and the survey ended.

#### 3.1.3 Questionnaires

The demographics questions assessed the gender and age of the participants as well as their responsibility for grocery shopping at least for most of their meals. Same as previous studies, participants’ environmental belief was assessed by NEP (Dunlap et al., 2000), which includes 15 items answered on a 5-point Likert-scale, and Health Consciousness (HC) was measured by 4 items rated on a 7-point Likert scale (Mai and Hoffmann, 2015). Four questions about consideration of the impact of plastic packaging were added according to Weber Macena et al. (2021) where participants were asked to rate the extent to which they think about the negative impact of plastic packaging on the environment on a 1–7 Likert scale.

#### 3.1.4 Evaluative conditioning

During the evaluative conditioning (EC) phase in both studies, positive information was provided for six product images and negative information for six other images. Moreover, six product images were not included in the conditioning phase at all in order to serve as control items. Each product image was shown for 1 s, followed by an affective climate image for 1 s. Then a fixation cross was shown with an inter-trial interval that varied between 800 and 1,200 ms before the next combination was shown. All combinations were shown three times, in three separate blocks (with a break in between) to ensure associative learning. In between every block, a grey circle was shown where the participant could choose to take a break and continue when they felt ready to do so. Before they could continue, a reminder was shown to evaluate the sustainability of the product packaging and not the product itself.

The affective image pairs used to facilitate the conditioning were the same in both studies. These were selected from the climate-related images gathered in Study 2. Six pairs (positive vs. negative) were selected for evaluative conditioning (Figure 6). The selection was based on participants’ ratings of the contextual pairing and how large the difference in valence ratings were between both images. In both studies, six products were conditioned with positive affect, six with negative affect and six products were paired to no image at all.

##### 3.1.4.1 Study 3: Extremely (un)sustainable

Study 3 focused on EC applied to “extreme” products, i.e., products where respondents had a strong opinion about the sustainability level of their packaging. Therefore, the top (Figure 3A) and bottom (Figure 3B) nine products in the sustainability ranking were selected. The nine product images with the highest sustainability ratings (*M* = 6.57, *SD* = 0.03) did not include any packaging, while the other nine products with the lowest sustainability ratings (*M* = 1.93, *SD* = 0.15) were all packed in more plastic than what is strictly essential for containing, transporting and preserving the product.

The EC phase of Study 3 thus comprised of six conditions. Of the nine product images with the highest sustainability ratings (i.e., products with no packaging), three were paired to a positively-valenced nature image, three were paired to a negatively-valenced image and three products were not paired at all. This led to three experimental conditions for sustainable products where sustainability was reinforced, sustainability attributes were weakened, or there was no conditioning (i.e., sustainability perception should not be changed). Similarly, for the nine product images with the lowest sustainability ratings (i.e., products with wasteful packaging), three were paired to a negatively-valenced image (i.e., wastefulness association was reinforced), three were paired to a positively-valenced image (i.e., wasteful association was weakened), and three products that were not paired at all (the attitude should not be changed as no evaluative conditioning was provided). Assigning a product image to an EC condition was performed randomly for every participant. Data was calculated per person and condition. This means that for every participant, their responses to each group of product images (Sustainable vs. Unsustainable packaging; three products each) receiving one of the three EC conditions (either Positive, Negative, or No Affect) were summarized.

##### 3.1.4.2 Study 4: Uncertain about sustainability

**Figure 8:**
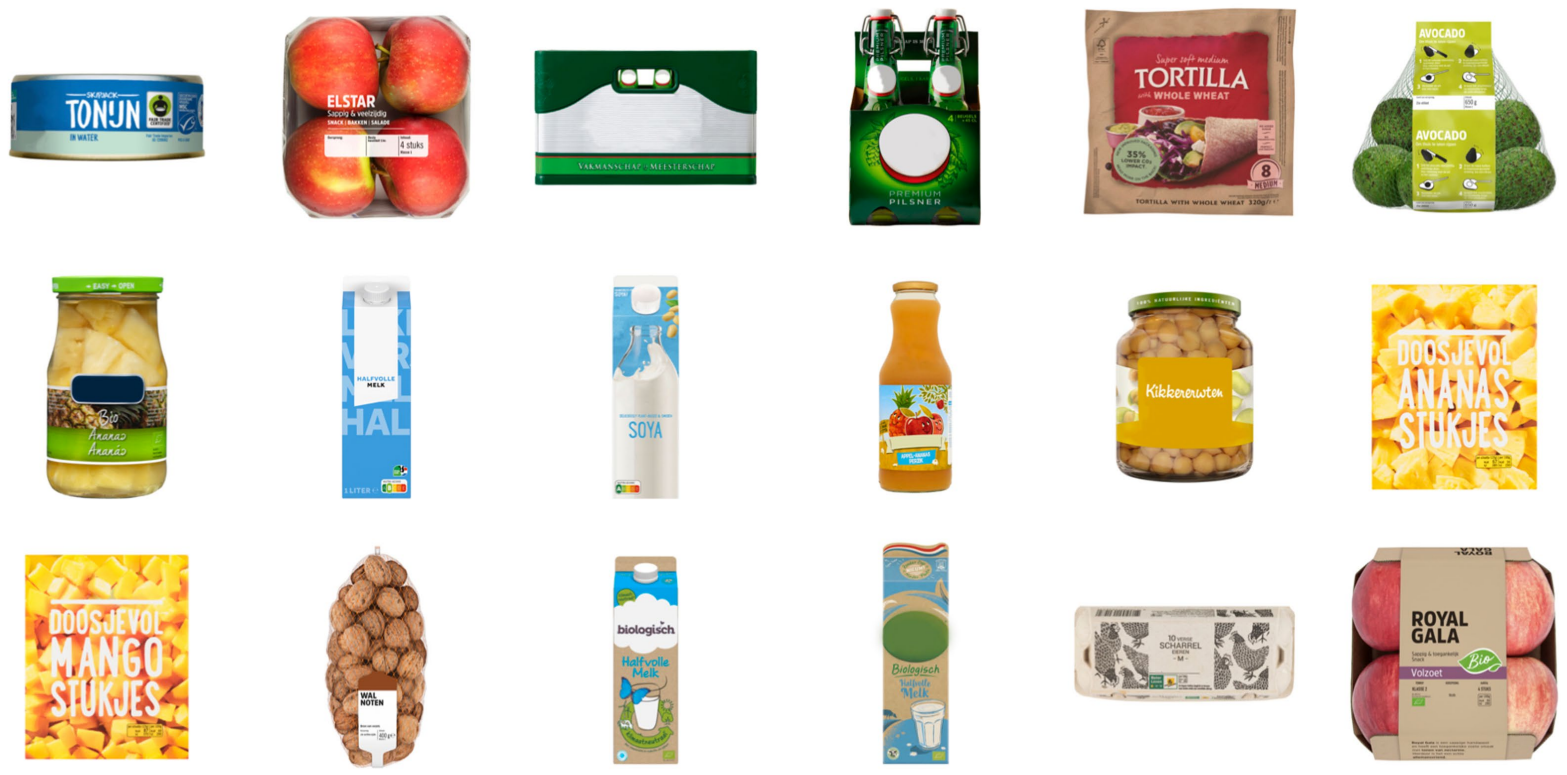
All product images that were used for evaluative conditioning in Study 4. They were rated to be neither unsustainable nor sustainable, indicating participants’ uncertainty about their sustainability qualities. Images reproduced with permission from Albert Heijn.

Study 4 focused on EC applied to product images where respondents were uncertain about the sustainability level of their packaging. Consequently, 18 product images were chosen such that their rating was around the middle of the scale (4 on a 1–7 Likert scale; *M* = 4.00, *SD* = 0.48, min = 3.19, max = 4.96) indicating that the majority of participants rated them as “neither sustainable nor unsustainable.” Their packaging was mostly recyclable, glass, beverage cartons or plastic nets (Figure 8).

Similar to Study 3, the EC phase comprised of three conditions. Six product images were paired to positively-valenced nature images, six other product images were paired to negatively-valenced images and six images were not paired at all. Assigning a product image to an EC condition was performed randomly for every participant. Data was calculated per person and condition. This means that for every participant, their responses to each of the three EC conditions (either Positive, or Negative, or No Affect; six products per condition) were summarized.

#### 3.1.5 Sustainability Association Task

The perception of sustainability and wastefulness of each product image was assessed before and after the EC phase with the Sustainability Association Task (SAT). This task is based on the presumption that the strength of an association between an object (i.e., product packaging) and an attribute (i.e., sustainability) is reflected in the participant’s response latency: when stimuli are easy to process (which is the case for objects and evaluations that are perceived to be congruent) participants respond faster to these stimuli (Fazio et al., 1986). Compared to the Likert scale, this may better represent the participants’ opinion or cognitive process (Fazio, 1990; Fulcher et al., 2016). These basic assessments of object-evaluation association (Fazio et al., 1986) provide the basis upon which implicit tests have been developed (Greenwald et al., 1998; Kardes et al., 2019) and is preferred for the measurement of attitude accessibility (Fazio et al., 1989).

**Figure 9:**
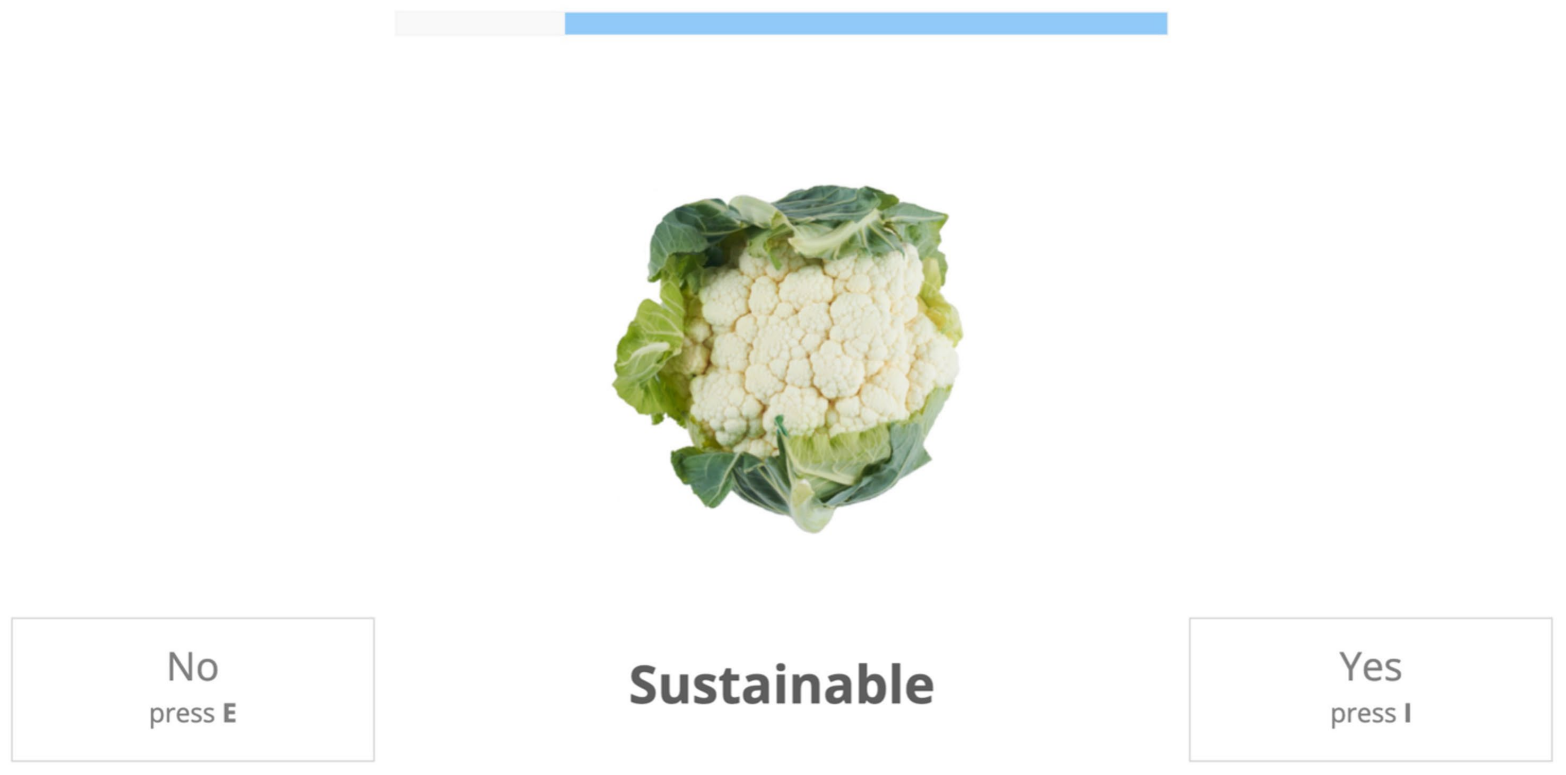
Example trial of the Sustainability Association Task (SAT). Eighteen products were presented with both words Sustainable and Wasteful (in total 36 trials). Participants had to press E (No) or I (Yes) within the time limit of 5 s to demonstrate their perception of the product. Images reproduced with permission from Albert Heijn.

The SAT consisted of the presentation of a product image with the words *Sustainable* or *Wasteful* (one at a time) underneath. Participants answered whether they thought these words fit the packaging of the product using E (*No*) and I (*Yes*) keys on the keyboard (see Figure 9). Participants had 5 s to answer each trial. The timer was shown on top of the screen as a blue bar that was filling up. If participants could not answer on time, the test moved to the next trial. Before the task, there was a practice block where participants could practice the task with two product images and two associations (*Sustainable* or *Wasteful,* four trials in total). The practice block is not included in the analysis. After the practice round, participants were once again presented with the task explanation and a reminder to focus on the packaging of the product. Afterwards, they were granted 5 s to place their fingers on the keyboard to start the task. The SAT task was executed two times, pre-EC and post-EC, to assess how evaluative conditioning would change the perception of sustainability for the product packaging. Participants were reminded to focus on the packaging of the product before every task. The procedure was identical in both pre-and post-EC tasks.

#### 3.1.6 Analysis of SAT responses

SAT responses were filtered when the response latency was below the lower boundary of 300 ms or above 5,000 ms as in Nosek et al. (2014). Participants were removed from the analysis completely if more than 10% of the SAT trials were filtered. Responses to trials with *Wasteful* association were re-coded such that *yes* meant unsustainable and *no* meant sustainable.

Data was summarized as one Associative Strength score per participant and condition, i.e., for each participant, we obtained scores that summarized their responses before and after conditioning per product category (Sustainable, Unsustainable, or Uncertain) and per conditioning type (Positive, Negative, No Affect). Moreover, for each participant, the average pre-EC Sustainability Rating was reported as a comparison of initial sustainability perceptions in all EC conditions. This Sustainability Rating is the percentage of trials in which the participant answered *yes* when the word *Sustainable* was shown and *no* when the word *Wasteful* was shown.

The Associative Strength is calculated according to Equation 1 and reflects the strength of the sustainability association by combining the explicit sustainability rating and its response latency into one comprehensive value. The yes percentage (%yes) reflects the Sustainability Rating. RT_yes_ and RT_no_ are the averaged response latencies corresponding to those trials, and RT_mean_ is the grand average of response latency over all trials in the pre-or post-EC tests for a specific participant. This grand averaging was employed in the equation to normalize the response latency values per participant and successfully reflect their variation between conditions and measurements, which is an important modulation in response latency research (Kardes et al., 2019). Response latencies (RT_yes_, RT_no_, and RT_mean_) were reversed in the equation such that a shorter reaction time indicated a stronger association.


(1) $$\mathrm{Associative}\;\mathrm{Strength}=\;\frac{\left({\%}_{\mathrm{yes}}\times \frac{1}{RT_{\mathrm{yes}}}\right)-\left({\%}_{\mathrm{no}}\times \frac{1}{RT_{\mathrm{no}}}\right)}{\frac{1}{RT_{\mathrm{mean}}}}$$

Consequently, the obtained Associative Strengths from the pre-EC SAT were subtracted from the post-EC scores such that the difference resulting from conditioning could be compared per participant.

#### 3.1.7 Statistical analysis

Associative Strength differences were compared in R (R Core Team, 2022) using repeated measures ANOVA from the R package rstatix (Kassambara, 2023) or Friedman Test from the stats package in case the assumption of homogeneity was not met following a Levene test. *Post-hoc* tests were done with paired *t*-tests or Wilcoxon Rank test when the data was not normally distributed following the Shapiro–Wilk test. *Post-hoc* tests assessed the change in Associative Strength caused by the conditioning in comparison to the NA conditioning and were corrected to a significance level of 0.025 following Bonferroni correction for two comparisons (Positive to NA and Negative to NA). Effect sizes were computed following Wilcoxon effect size from rstatix (Kassambara, 2023). Also, dplyr (Wickham et al., 2023) and ggplot2 (Wickham, 2016) packages were used for data exploration and visualization.

## 4 Results

From the SAT tasks in Study 3 and 4, two metrics are reported: the Sustainability Rating and the Associative Strength. The Sustainability Rating is evaluated before the EC to validate the initial product packaging perceptions. Afterwards, the difference in Associative Strength in response to the product images is reported, which is an implicit measure reflecting how strongly the product packaging was associated with sustainability. The raw SAT responses of each test are reported in the Supplementary material.

### 4.1 Sustainability Rating

**Figure 10:**
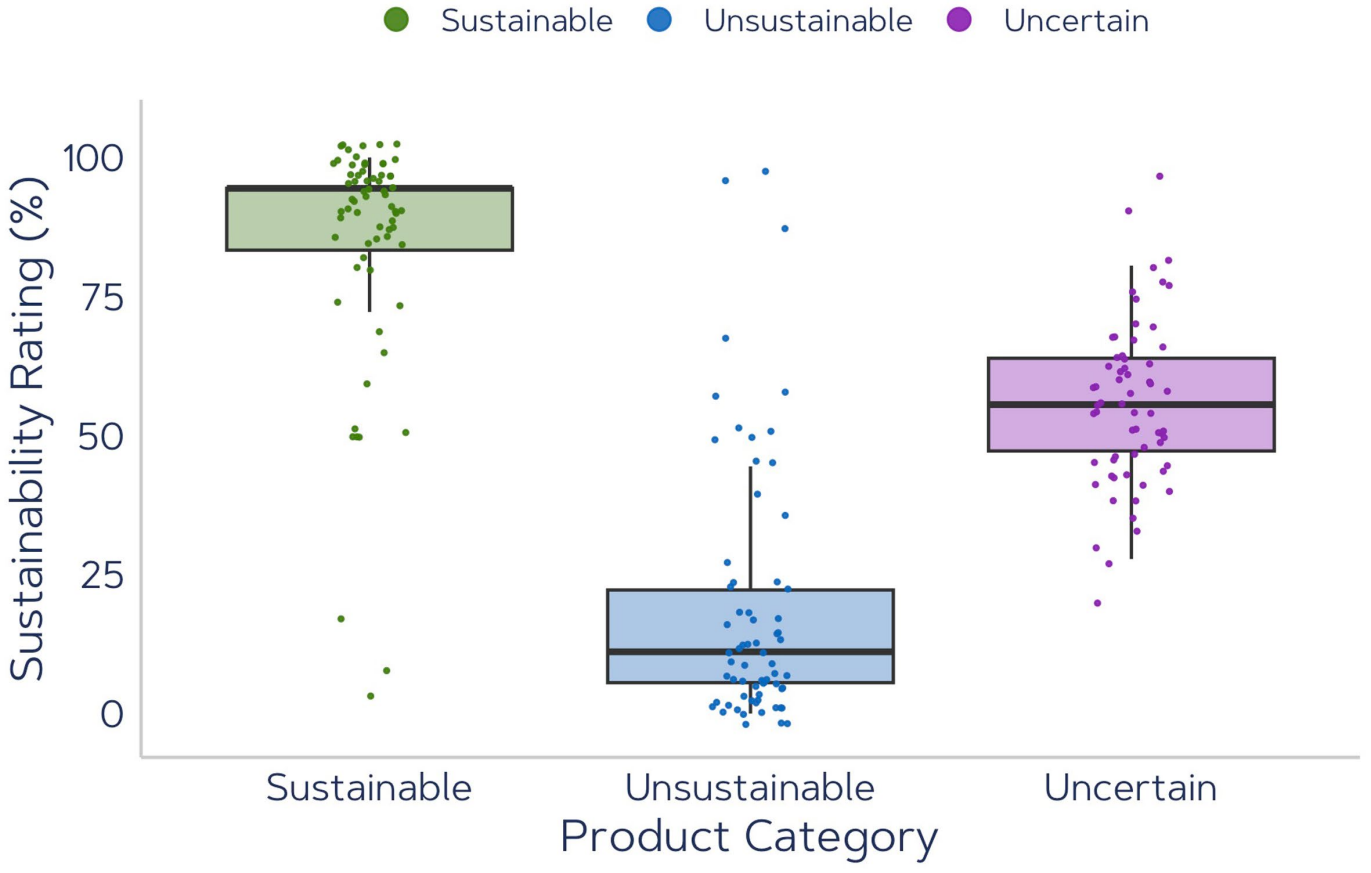
Averaged Sustainability Ratings per participant in pre-EC SAT for Sustainable products (average of nine sustainable products with two associations), Unsustainable products (average of nine unsustainable products with two associations), and Uncertain products that were rated neither sustainable nor unsustainable (average of 18 products with two associations).

The Sustainability Rating is the number of times the participant responded that a product is sustainable: either by answering *yes* when the attribute *Sustainable* was shown or *no* when the attribute *Wasteful* was presented. This metric is an explicit measurement of participants’ opinion but since time pressure was added to the trial, the response alone could be an indirect measurement of the participant’s attitude. Figure 10 demonstrates the Sustainability Ratings for product images with Sustainable, Unsustainable and Uncertain packaging categories before EC. As was expected and can be seen in Figure 10, for the products in the Sustainable and Unsustainable categories, the pre-EC SAT test showed considerably strong opinions: the median of sustainability ratings was at the top of the scale and at the bottom for the sustainable and unsustainable product categories, respectively. For the Uncertain product category, the median was around the middle, indicating that most people rated some of the product images as sustainable and a similar number of images as unsustainable. This reflects that the sample in this study has similar sustainability perceptions of the products compared to the participants in Study 1.

### 4.2 Associative Strength

**Figure 11:**
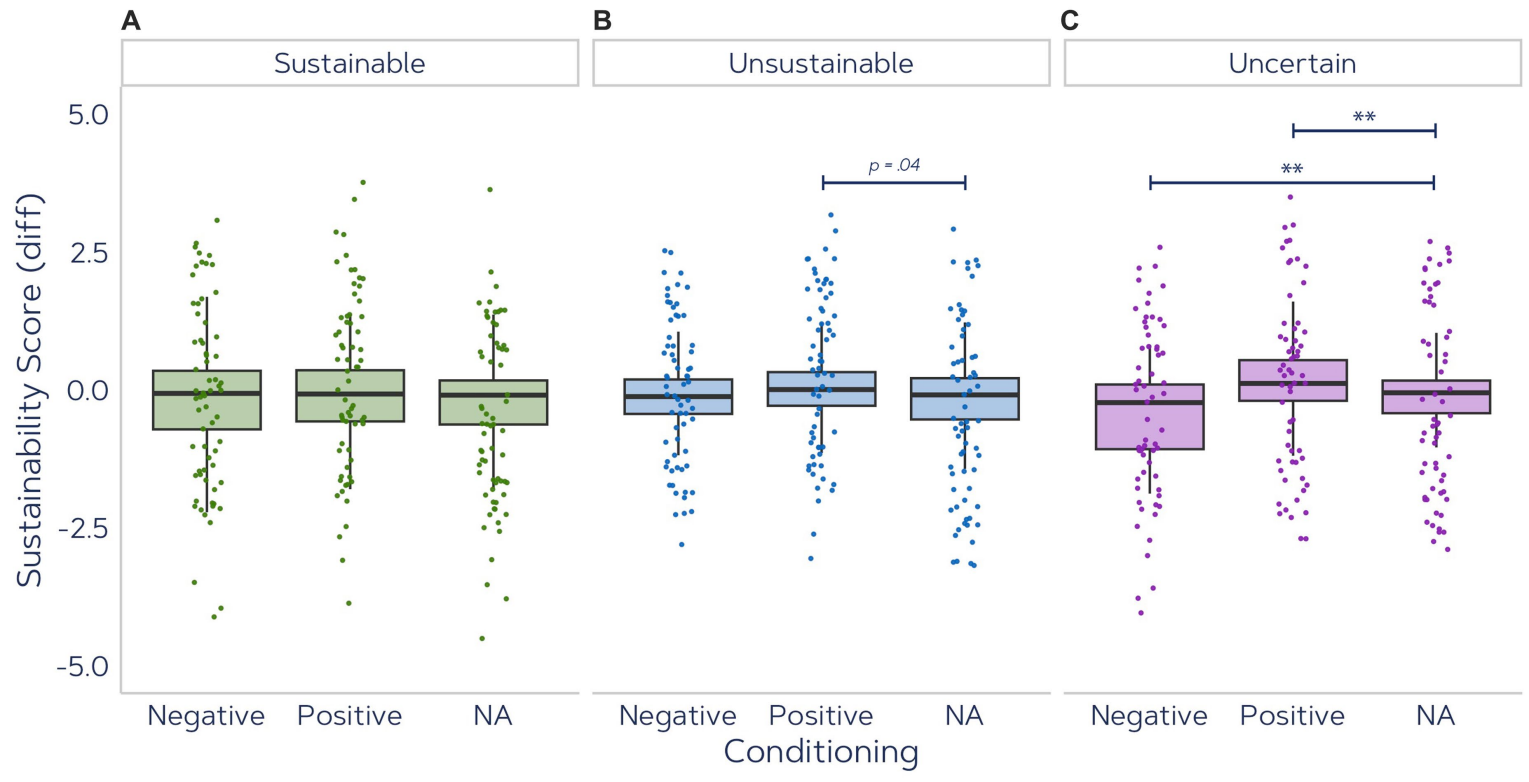
Averaged Associative Strength per participant for each condition in pre-and post-EC SAT tests for **(A)** Sustainable products, **(B)** Unsustainable products, and **(C)** Uncertain products that were rated neither sustainable nor unsustainable (**p < 0.01, *p < 0.025, *p*-values <0.05 are indicated with rounded values).

The Associative Strength reflects the strength of an implicit response by incorporating the response latency into the explicit sustainability rating according to Equation 1. The Associative Strength differences between pre-EC and post-EC for all product categories (Sustainable, Unsustainable and Uncertain packaging) are displayed in Figure 11. For the Sustainable product category, Associative Strength did not show significant variations between Positive vs. Negative vs. No Affect (no conditioning) at all [*F*(2,130) = 1.754, *p* = 0.177].

For Unsustainable packaging category, a significant main effect was observed [*F*(2,130) = 3.891, *p* = 0.023, $\eta^{2}$ = 0.014]. However, post-hoc analysis between Positive EC (*Mdn* = −0.120, *IQR* = 0.625) and NA conditioning (*Mdn* = −0.089, *IQR* = 0.748) did not show significantly different changes (*W* = 776, *p* = 0.036), nor did comparison of Negative EC (*Mdn* = 0.007, *IQR* = 0.609) to NA conditioning (*W* = 1,269, *p* = 0.298).

For the Uncertain category, the change in Associative Strength scores was significantly impacted by the type of conditioning [ $\chi^{2}$ (3) = 119.79, *p* < 0.001]. Positive EC (*Mdn* = 0.119, *IQR* = 0.737) compared to NA (*Mdn* = −0.051, *IQR* = 0.592) induced significantly greater change in Associative Strength (*W* = 508, *p* = 0.002, *CI* = [0.11, 0.46], *r* = 0.24). Similarly, Negative EC (*Mdn* = −0.229, *IQR* = 1.17) significantly reduced the sustainability association (*W* = 1,340, *p* = 0.005, *CI* = [−0.50, −0.09], *r* = 0.22).

### 4.3 Individual differences in conditioning effect

The previous analyses seem to indicate that evaluative conditioning affected the perception of packaging sustainability depending on the product category (Sustainable, Unsustainable, Uncertain) and conditioning direction (Negative, Positive, NA). The following analysis aims to investigate individual factors (such as gender, age, NEP, and Health Consciousness; HC) that could impact one’s predisposition to conditioning effects and hence the change in Associative Strength after conditioning.

The average NEP was 3.58 (*SD* = 0.44) in Study 3 and 3.51 (*SD* = 0.51) in Study 4. Cronbach’s alpha for this 15-item questionnaire was 0.68 in Study 3 and 0.81 in Study 4. The average HC in Study 3 was 5.30 (*SD* = 0.94) and 5.22 (*SD* = 0.72) in Study 4. Cronbach’s alpha for this 4-item questionnaire was 0.66 in Study 3 and 0.55 in Study 4. The impact of plastic according to the participants in Study 3 was 5.10 (*SD* = 1.13) and 4.88 (*SD* = 1.14) in Study 4. Cronbach’s alpha for 4-item questionnaire was 0.76 in Study 3 and 0.76 in Study 4.

For gender, a comparison of means was done for each conditioning type. Effects of age, NEP and HC were compared with linear regression. However, most personal factors had no effect on the conditioning results. NEP (*M* = 3.59, *SD* = 0.44, on a 1–5 Likert scale) and age (*M* = 20.43, *SD* = 2.94) did not impact the difference in Associative Strength between conditioned stimuli in a linear or quadratic way. Gender, too, did not have any impact on conditioning effects, nor did NEP scores vary significantly between genders.

The only individual factor that was related to EC effect on Associative Strength was HC: for all products together, higher HC scores pointed towards decreased differences in Associative Strength after positive conditioning (*R^2^ adj* = 0.01, *p* = 0.050, *CI*_Intercept_ = [0.02, 1.27], *CI*_HC_ = [−0.24, 0.00]), but not after negative conditioning. This indicates that individuals who are more conscious of their health (as compared to individuals lower on HC), tended to show a greater increase in sustainable attitude after the stimuli were conditioned with images showing positive affect. The HC scores tended to be slightly higher for females than males (*W* = 1,687, *p* = 0.056).

### 4.4 Discussion

The results of Study 3 and 4 show that evaluative conditioning with climate-related affective images can be effective, especially for products with packaging that has ambiguous sustainability quality. This indicates that products for which consumers do not have a strong opinion can be effectively conditioned to be perceived as more or less sustainable. Pairing highly sustainable product images with positive images of nature and highly unsustainable product images with negative images of climate change did not change participants’ perception of the product’s sustainability. However, positive and negative conditioning of products with uncertain packaging qualities yielded significant changes in the hypothesized direction. There was also a slight trend for products with unsustainable packaging that were paired with positive images to be perceived as slightly less unsustainable.

Additionally, these effects of EC were not modulated by individual factors. Only health consciousness was an important factor for stronger conditioning with positive images. We initially hypothesized that NEP could have a quadratic effect on Associative Strength values, in the sense that individuals with low environmental belief would not be impacted by climate change images as they do not really care for the environment, whereas individuals with high NEP score would be better informed and thereby not impacted by (false) information provided in the experiment. However, we did not observe a quadratic or linear relationship in the data.

These results suggest that when designing evaluative conditioning interventions, one should pay attention to the observable (sustainability) attributes of the product and its packaging as the intervention might not be effective for highly evident ones. We also observed in our study that conditioning of highly sustainable products with a positive image did not yield more positive reactions in the post-EC test. The fact that the attitude towards extremely (un)sustainable product packaging is not easily changed was expected given the finding by Hofmann et al. (2010) that ambiguous stimuli yield stronger EC effects than strongly valued ones.

## 5 General discussion

The goal of this research was to examine the effectiveness of evaluative conditioning as a potential tool for future pro-environmental attitude and behavior change interventions. We conducted four studies in which we first compiled two validated datasets of supermarket products and climate-related affective images, and then used them to measure how coupling of affective images of nature and climate impact with product images can change people’s perception of their (un)sustainability. Our results confirm our hypothesis that providing information about a product packaging’s environmental impact using evaluative conditioning method can change people’s perception towards that product image, although this effect is dependent on how the packaging was perceived in terms of sustainability before conditioning. Especially when there is no consensus about the sustainability of the product image (as was the case with the Uncertain products in Study 4), conditioning using affective images of climate change could induce a considerably strong effect in changing people’s attitudes towards it.

As our results from Study 3 show, both positive and negative EC did not have a significant effect on the perception of the product image, indicating that EC is not effective when a strong opinion about the product image already exists. Importantly, a trend was observed that positive information about unsustainable product images might slightly improve the sustainability perception of that image, while this was not the case with sustainable product images that were paired with negative affect. This was surprising at first because a stronger conditioning effect of negative images was expected as negative climate images can elicit higher arousal (Lehman et al., 2019), which we also observed in Study 2. However, the results did not show a stronger effect with negative images compared to positive ones for the uncertain product images in Study 4, and in Study 3 we found the opposite of the hypothesized direction: the positive images tended to change the perception of unsustainable product images while the negative images could not change the perception of sustainably rated product images. In our view, there could be several explanations for this observation: (1) the positive images are stronger in manipulation of the sustainability attitude than the negative images, although this is unlikely as in that case we would have observed a stronger effect with positive images for the uncertain products as well; (2) the attitude towards sustainable products is more robust than the attitude towards the unsustainable product category, which could also be a likely hypothesis when looking at Figure 10; (3) participants are more susceptible to receive positive information about unsustainable products than negative information about sustainable products. This last explanation could be interpreted in the context of motivated reasoning: participants might be more prone to accept contradicting information about unsustainable product images because it is more in line with their pre-existing beliefs or motivations (Palminteri, 2023). This is especially important to consider when referring to greenwashing: information about the negative consequences of a choice might be less likely to stick with the individual than the positive consequences of a decision.

Following the results of Carlson et al. (2019) who showed that images of climate change attract attention, especially in individuals with an environmental predisposition, we hypothesized that individuals with higher NEP scores would show a significantly larger change of sustainability ratings after negative conditioning. Previous studies showed that the effect of evaluative conditioning was stronger in participants who were on the extreme opposite of the conditioning direction. For example, Hollands et al. (2011) found that EC for healthy eating was only effective in individuals that were really unhealthy. Similarly, Koenig-Lewis et al. (2022) observed that individuals with higher health consciousness were not sensible to additional health motivation cues, whereas individuals with a lower consciousness of their health were highly stimulated by EC. However, we did not observe any effect of environmental predisposition on EC effects in our sample.

### 5.1 Limitations

In comparing the effect of conditioning on both product types (products with extreme and uncertain sustainability ratings), it is important to note they were tested in separate studies, which could impact the results. Testing the product groups separately was done to reduce the length of the study and ensure respondents were able to keep their attention. Additionally, this meant that the scores in the extreme (un)sustainable products were derived from three products averages (compared to six with the uncertain ones). It may have been the case that the discrepancy between obviously (un)sustainable made it easier for respondents to recall their initial response, or they denied the conditioning as they already felt a strong opinion about the products.

To measure individual factors, we relied on existing questionnaires in the literature. There is a wide variety of measurements that categorize participants in pro-environmental groups. According to Fishbein and Ajzen (2010) and the principle of compatibility, behavior can only be predicted by measures that are aimed at the same level of specificity (i.e., abstract behavior can only be assessed by abstract measurements). Since there are several measurements for environmental attitude, belief, and behavior, it might be that NEP is not the most compatible when correlated with the sustainability perception of daily products. Moreover, pro-environmental tendencies that occur automatically may be modified or overridden by slower reflective reasoning (Fazio and Olson, 2003), indicating that more implicit measurements of pro-environmental tendencies are perhaps better indicators of attentional processes than the explicit counterparts used in this study (Meis-Harris et al., 2021). Still, NEP is considered a powerful predictor of environmental concern (Xiao et al., 2019). Scholars agree that it measures the basis of ecological beliefs, and studies typically find that the NEP has considerable power in predicting pro-environmental behaviors (Xiao et al., 2019).

The effect sizes found for the conditioning of uncertain products were lower than the minimum detectable effect size calculated by the sensitivity analysis, which means that these results must be interpreted with caution. The observed effect sizes were also lower than the effect sizes reported by Hollands et al. (2011), which were used for *a priori* determination of sample size. The larger effect size in health conditioning performed by Hollands et al. (2011) indicates stronger conditioning effects for health-related problems that are more psychologically tangible and relatable to individuals whereas climate change is still an abstract and somewhat distant problem to many people.

Moreover, the longevity of the effects of the intervention could not be established from the current study, as the attitude change was only measured directly after the intervention. Previous studies have indicated that conditioning might have effects that exceed the duration of the experimental session: one (Houben et al., 2010) or two (Meijers et al., 2021) weeks after the intervention, effects were still observed. Especially when the affective combination is repeated multiple times (Hofmann et al., 2010), the effects may last beyond the duration of the experimental session, but longitudinal evidence should be collected.

### 5.2 Future research

While the results of our experiments indicate an effect of climate change images on people’s perception of supermarket products (particularly in the context of product packaging and sustainability), it is widely debated whether such climate change images are appropriate for behavior change interventions. Studies have suggested that the pictures containing climate disasters could be depressing and more likely to lead to psychological distancing (Leviston et al., 2014) and in-action (Schneider et al., 2017; Hornsey and Fielding, 2020). Meis-Harris et al. (2021) observed that individuals who actively engaged in pro-environmental behavior were paying more attention to environmentally harmful objects such as plastic bags, but not to beneficial objects such as reusable bags. However, other studies showed the opposite. Namely, for individuals who already engage in pro-environmental behavior, positive images of climate change solutions (such as solar panels) tend to capture more attention than negative images displaying climate change disasters (Carlson et al., 2020; Meis-Harris et al., 2021; Carlson et al., 2022). These inconsistent findings in previous research warrant more research to improve our understanding of individuals’ susceptibility to various climate-related images and their effects on emotional responses. Images of climate change solutions were not included in the stimulus set proposed by this study but could be an interesting option for future research when investigating evaluative conditioning for pro-environmentally oriented individuals. Moreover, future research may consider increasing the variability of climate change images in the stimuli set to improve the categorical representation of positive and negative affect, which could increase the generalization of the conditioned response towards novel instances (Reichmann et al., 2023).

Our findings suggest that evaluative conditioning could change people’s assessment of supermarket products before and after coupling with affective climate-related images, but future research is still required to examine how this change in perception is reflected in daily behavior. Additional questions for future research are why climate change images seem to be working and how their potential for pro-environmental behavior change interventions can be harnessed. In the current study, we could not identify all the relevant factors on an individual level that could lead to a predisposition to conditioning effects. Since we are not yet at a point where all individuals are considerate of the environment (Weckroth and Ala-Mantila, 2022), an approach that appeals to individuals with a lower concern for the environment might be more effective in promoting pro-environmental behavior among them. This calls for a personalized approach that captures individual attitudes, motivations and socioeconomic factors as well.

To dive deeper into the dynamics of individual factors, additional insight into the cognitive, emotional, and neurophysiological components of pro-environmental behavior and attitudes could be important (Leviston et al., 2014; Van Cappellen et al., 2018; Doell et al., 2021). Several studies have shown that emotional reactions to climate change play a part in pro-environmental behavior. However, next to the emotional component, cognition is also shown to be important. For example, memory or attention can be predictors of the EC effect (Corneille and Stahl, 2019), as well as mood and motivations (Sperlich and Unkelbach, 2022). Carlson et al. (2022) presented a working model for climate change psychology, arguing that interventions can be most effective in mitigating climate change behavior, when they also target the neural circuitry underneath. The same line of research was proposed by Leeuwis et al. (2022b) who recommended the investigation of the neural dynamics underlying pro-environmental motivations and behavior as a target metric for intervention design.

The positive results in conditioning observed here lead to the hypothesis that targeting interventions at an implicit level indeed may have an effect on cognitive and emotional drivers of pro-environmental attitudes and behaviors. Thus, to further investigate the cognitive and emotional components underlying evaluative conditioning for pro-environmental behavior change, we propose for future research to apply neurophysiological measurements to the proposed framework. For example, Bosshard et al. (2019) conditioned (dis-)liked brand names with (un-)pleasant sounds and observed that while there were no changes in explicit liking of the brands, variations were observed in neural measures: EEG frontal asymmetry increased for disliked brands when coupled to pleasant sounds and similarly decreased for liked brands that were coupled to unpleasant sounds. Moreover, differences in event-related potentials were observed after extensive sessions of evaluative conditioning (Kuchinke et al., 2015). Other metrics could focus on reward, valence, motivation, and engagement (Michaelsen and Esch, 2021; Sawe and Chawla, 2021; Leeuwis et al., 2022a).

Next, to the individual factors that play a role in pro-environmental attitudes and behavior, social factors could be further explored by (experimental) social psychology. For example, social influences and in-group norms have a strong effect on PEB (van Riper et al., 2019; Bouman et al., 2021; Cialdini and Jacobson, 2021). Strong individual evaluations may generalize to a social group (Moran et al., 2023), making it interesting to investigate the effect of evaluative conditioning in (several) members of a group.

The line of research into (evaluative) conditioning is still developing. As Moran et al. (2023) reviewed, some boundary conditions for successful conditioning are clear. For example, the evidence is clear that contingency awareness is essential for EC to be successful. This means that EC requires the participant to consciously notice the pairing of images (conditioned stimulus and unconditioned stimulus), but for EC to have effect, the participant does not need to be aware of the change in valence towards the conditioned stimulus after EC. To further understand the limits and possibilities of evaluative conditioning, more research in various application areas is needed, both tested inside and outside of the lab.

### 5.3 Practical implications

Since this study taps into real-life problems such as climate change, excessive consumption, and plastic waste, the implications beyond academic literature should also be discussed. Communicators such as journalists and scientists should be very careful with the information they distribute. False information may cause a (small) opinion shift in sustainability perception among consumers within only three repetitions. This is important to keep in mind when looking at the fake news movements.

This line of research could lead to the development of shopping and entertainment applications where emotional conditioning is used as a key asset in moving consumers towards more pro-environmental behavior. Nature images provide a strong cue in green advertising (Hartmann et al., 2013, 2023), making it an attractive asset for advertisers. For example, online social media could be used to pair products and images the same way it is implemented in an evaluative conditioning paradigm (Moran et al., 2023).

However, consumers and companies should be cautious about false communications and greenwashing. Greenwashing refers to the act of communicating sustainability qualities when the product (packaging) is indeed not (Walker and Wan, 2012). Next to the issue of morality and consumer misinformation, greenwashing has negative effects on the instances employing it; the inconsistency between a (falsely) green attribute of a product and the accompanied higher price may have a negative effect on the consumers (Lee et al., 2018). Hence, green marketers should stay true to their mission when implementing evaluative conditioning. Simultaneously, evaluative conditioning could also be used as a tool to educate consumers on the true impact of certain products, packaging types or claims, in this way combatting greenwashing practices. Although, ways to do this still need to be further investigated (Volschenk et al., 2022; Álvarez-García and Sureda-Negre, 2023).

The environmental impact of packaging is still growing, but technology that is currently available can be useful to redesign the packaging paradigm (Escursell et al., 2021). We would like to encourage packaging researchers to further dive into the possibility of more sustainable production and material adoption. Meanwhile, the academic field of social psychology and cognition may focus on investigating the drive for consumers to make use of it.

## 6 Conclusion

This work presents the kick-off for further research into the affective underpinnings of pro-environmental behavior. In order to investigate the change in the sustainability perception of products that a consumer encounters daily, an openly accessible stimulus set in Study 1 was created containing 94 product images that were rated on the sustainability of their packaging. This showed that the most sustainable products were the ones that did not have any packaging at all, while the most unsustainable products were packed in an unnecessary amount of plastic. In Study 2, another openly available database was created with images of climate change and nature that were rated based on their relevance to climate change, arousal, and valence of response induced in the participants. Combining these two image sets, Study 3 and 4 aimed to investigate how these affective images could be implemented in an evaluative conditioning intervention to change the perception of product sustainability in participants. Our results showed that for products where participants were uncertain about the sustainability of their packaging, both negative and positive conditioning had a strong effect in changing the sustainability association toward the intended direction. However, for products with clearly sustainable or unsustainable packaging, the effect was negligible. These results provide ground for future research to further investigate the emotional dynamics that govern pro-environmental attitude and behavior. Additionally, by sharing the stimulus sets with the scientific community, we aspire to contribute to the open science movement in order to expedite efforts for mitigating the climate change problem.

## Data availability statement

The original contributions presented in the study are included in the article/Supplementary material, further inquiries can be directed to the corresponding author.

## Ethics statement

The studies involving humans were approved by Research Ethics Committee of Tilburg School of Humanities and Digital Sciences. The studies were conducted in accordance with the local legislation and institutional requirements. The participants provided their written informed consent to participate in this study.

## Author contributions

NL: Conceptualization, Formal analysis, Methodology, Visualization, Writing – original draft, Writing – review & editing. TB: Conceptualization, Supervision, Writing – review & editing. MT: Writing – review & editing. MA: Conceptualization, Supervision, Writing – original draft, Writing – review & editing, Methodology.

## Footnotes

^1^ https://www.ah.nl/producten
