# Supplementary material for: Uncovering the potential of evaluative conditioning in shaping attitudes toward sustainable product packaging
Source: Front Psychol. 2024 Mar 14;15:1284422. doi: 10.3389/fpsyg.2024.1284422 (PMC10977460; doi:10.3389/fpsyg.2024.1284422)
Supplement: Supplementary file 1 [file Data_Sheet_1.docx]

# Supplementary Material

Accompanying this work, two stimulus sets were created that are openly accessible. Supplementary material A contains the images of supermarket products and their sustainability ratings, supplementary material B contains the affective images of nature and climate change impact together with their ratings on relevance to climate change, arousal and valence.

## Supplementary material A

The product images and their sustainability ratings could be found here:

<https://osf.io/bc7ms/?view_only=59f6f0bd7f364f42ba5f9d6c6f4c5cad>

## Supplementary material B

The climate change images and their ratings on relevance, arousal and valence can be found here:

<https://osf.io/s52g7/?view_only=b66f29e4d1e147c292f2e0d57736e828>

## Supplementary material C

The responses and response latencies of each subject in Study 3 and Study 4 can be found here:

<https://osf.io/4vnx2/?view_only=3724e52d75d748828278ec7f22cf18f8>
